# Evaluation of the safety and potential lipid-lowering effects of oral hydrogen-rich coral calcium (HRCC) capsules in patients with metabolic syndrome: a prospective case series study

**DOI:** 10.3389/fnut.2023.1198524

**Published:** 2023-07-14

**Authors:** Szu-Han Chiu, Frank L. Douglas, Jia-Ru Chung, Kuang-Yih Wang, Chao-Fang Chu, Hsia-Yun Chou, Wei-Chih Huang, Tian-Yu Wang, Wen-Wen Chen, Min-Chung Shen, Feng-Cheng Liu, Po-Jen Hsiao

**Affiliations:** ^1^Division of Endocrinology and Metabolism, Department of Medicine, Armed Forces Taoyuan General Hospital, Taoyuan, Taiwan; ^2^HOHO Biotech Co., Ltd., Taipei, Taiwan; ^3^HoGo Force Co., Ltd., Taipei, Taiwan; ^4^Department of Nursing, Min-Sheng General Hospital, Taoyuan, Taiwan; ^5^Rheumatology/Immunology and Allergy, Department of Medicine, Armed Forces Taoyuan General Hospital, Taoyuan, Taiwan; ^6^Rheumatology/Immunology and Allergy, Department of Medicine, Tri-Service General Hospital, National Defence Medical Center, Taipei, Taiwan; ^7^Division of Nephrology, Department of Internal Medicine, Taoyuan Armed Forces General Hospital, Taoyuan, Taiwan; ^8^Division of Nephrology, Department of Internal Medicine, Tri-Service General Hospital, National Defence Medical Center, Taipei, Taiwan; ^9^Department of Life Sciences, National Central University, Taoyuan, Taiwan

**Keywords:** metabolic syndrome, low-density lipoprotein (LDL) cholesterol, high-density lipoprotein (HDL) cholesterol, triglycerides, molecular hydrogen, hydrogen-rich coral (HRCC)

## Abstract

**Background:**

Metabolic syndrome is characterized by a cluster-like occurrence of conditions such as hypertension, hyperglycaemia, elevated low-density lipoprotein (LDL) cholesterol or triglycerides (TG) and high visceral fat. Metabolic syndrome is linked to the build-up of plaque within the artery, which leads to disorders of the circulatory, nervous and immune systems. A variety of treatments target the regulation of these conditions; nevertheless, they remain dominant risk factors for the development of type 2 diabetes (T2DM) and cardiovascular disease (CVD), which affect 26.9% of the US population. Management and intervention strategies for improving cholesterol and/or TG are worthwhile, and recent studies on hydrogen treatment are promising, particularly as molecular hydrogen is easily ingested. This study aimed to investigate the lipid-lowering effects and quality of life (QOL) improvement of hydrogen-rich coral calcium (HRCC) in patients with metabolic syndrome.

**Methods:**

The patients, all Taiwanese, were randomly assigned to 3 different doses (low, medium, and high) of HRCC capsules. The primary outcome was the adverse effects/symptoms during this 4-week use of HRCC capsules. The secondary outcome was lipid profile changes. Complete blood count, inflammatory biomarkers, and QOL were also measured before and after the course of HRCC.

**Results:**

Sixteen patients with metabolic syndrome completed this study (7 males, 9 females; mean age: 62 years; range: 32–80). No obvious adverse effects were recorded. Only changes in blood TG reached significance. The baseline TG value was 193.19 μL (SD = 107.44), which decreased to 151.75 μL (SD = 45.27) after 4 weeks of HRCC (*p* = 0.04). QOL showed no significant changes.

**Conclusion:**

This study is the first human clinical trial evaluating HRCC capsules in patients with metabolic syndrome. Based on the safety and potential TG-lowering effects of short-term HRCC, further long-term investigations of HRCC are warranted.

**Clinical trial registration:**

[ClinicalTrials.gov], identifier [NCT05196295].

## 1. Introduction

Metabolic syndrome is a group of conditions that includes risk factors such as obesity, elevations in blood pressure, blood triglyceride (TG), and fasting glucose levels, and low high-density lipoprotein cholesterol (HDL-C) (1). Together, these conditions raise our risk of coronary heart disease, type 2 diabetes (T2DM), stroke, and other serious health problems (2). The first-line treatment for metabolic syndrome includes weight reduction and increased exercise. Nevertheless, it can be difficult to implement exercise in daily life. Genetic predisposition also plays a role in the development of these risk factors (1). Diseases such as T2DM and cardiovascular disease (CVD) promote a pro-inflammatory state and require therapeutic attention. Drug targeting metabolic syndrome focus on reducing blood pressure and blood glucose levels, and include medications such as angiotensin-converting enzyme (ACE) inhibitors (such as Capoten and Vasotec) for blood pressure, cholesterol medications (3) such as statins, niacin, and Zetia, medications for treating diabetes, such as metformin (Glucophage), pioglitazone (Actos), and rosiglitazone (Avandia) (4), and low-dose aspirin, which can reduce the risk of heart attack and stroke. Although niacin and fibrates are observed to raise HDL-C and reduce TG and non-HDL-C concentrations, they modestly (if at all) reduce low-density lipoprotein cholesterol (LDL-C) (5).

The development of metabolic syndrome is primarily related to insulin resistance (6), in which insulin is inefficiently used to lower glucose and triglyceride levels. The conditions that characterize Metabolic syndrome result from an increase in hormones induced by free fatty acids (7), which are stored in adipose cells, and can influence how the body controls blood glucose levels (7). Inflammation is a risk factor for insulin resistance, high blood pressure, and diseases of the heart and blood vessels (8). In the presence of excess adipose tissue, immune cells interact with fat cells to produce pro-inflammatory cytokines, such as tumor necrosis factor α (TNFα) and interleukin-6 (IL-6), which promote an inflammatory state. These cytokines can cause plaque, a waxy substance, to build up inside blood vessels and obstruct blood flow. Individuals with Metabolic syndrome present with a pro-inflammatory state, which is recognized by elevated concentrations of C-reactive protein (CRP) levels (9, 10). CRP is observed to rise as the number of conditions associated with metabolic syndrome increases, and is elevated in obese patients who experience a corresponding decrease in CRP with weight loss (9). CRP has also been associated with future cardiovascular events (10). A higher erythrocyte sedimentation rate (ESR) is also associated with the development of metabolic syndrome and obesity (11), and a higher eosinophil count is associated with metabolic, cardiac, and pulmonary outcomes (12). Therefore, targeting the inflammatory response and lipid metabolism is an important strategy for treating metabolic diseases (13). The white blood cells (WBCs) are observed to increase significantly in patients with metabolic syndrome. A high WBC count positively correlates with insulin resistance and TG levels and is negatively associated with HDL-C (14). Thus, comprehensive plasma profiling can improve understanding on the overall risk factors for developing metabolic syndrome.

Molecular hydrogen is able to regulate inflammatory processes involved in cytokine and lipid metabolism (13). Kamimura et al. (15) demonstrated that molecular hydrogen reduces hepatic oxidative stress and alleviates fatty liver in db/db mice and fatty liver induced in wild-type mice by a high-fat diet. In patients with T2DM or impaired glucose tolerance (IGT), hydrogen-rich water (HRW) intake was associated with a trend toward decreased serum concentrations of oxidized LDL-C and free fatty acids and increased adiponectin and extracellular-superoxide dismutase (16). The levels of modified LDL-C (i.e., modifications that increase the net negative charge of LDL-C), small dense LDL, and urinary 8-isoprostane significantly decreased by 15.5% (16). In another report, the use of HRW decreased serum LDL-C and improved HDL function in patients with potential metabolic syndrome (17). Furthermore, LeBaron et al. (18) demonstrated that high-concentration HRW improves several biomarkers of cardiometabolic health in middle-aged men and women with metabolic syndrome, including body mass index, waist-hip ratio, resting heart rate, blood lipids, blood glucose, inflammation, and redox homeostasis. Recent research found reduced lipogenesis and enhanced lipolysis in the liver of rats exposed 2 h daily to hydrogen gas (H_2_) either by drinking HRW or by inhaling 4% H_2_ gas, a finding that was associated with a loss in visceral fat and brown adipose tissue and a reduction in serum lipids (19). Beyond regulating inflammation and lipid metabolism, hydrogen treatments are also shown to affect hypertension (20) and regulate the homeostasis in the cardiovascular system and related metabolic activities. Sugai et al. (20) showed in an animal model that H_2_ significantly suppresses increases in blood pressure after 5/6 nephrectomy. Molecular hydrogen has also been used to improve pulmonary hypertension (PAH) in a rat model by suppressing macrophage accumulation, reducing oxidative stress, and modulating the STAT3/NFAT axis, the latter which are important transcription factors associated with the immune response (21). Hydrogen supplements are available in three forms: encapsulated molecular hydrogen, H_2_ inhalation, and HRW. As H_2_ inhalation and HRW are well-established resources for ameliorating conditions induced by dysregulations in lipid and glucose metabolism, attention has been shifted to the potential of molecular hydrogen in managing diabetes mellitus, inflammation and cardiovascular health (22). Recent research has shown improvements in T2DM through the inhibition of oxidative stress which involves mechanisms that are associated with the toll-like receptor 4 (TLR4), pathogen recognition, the myeloid differentiation primary response 88 (MyD88), signaling within immune cells, and the NF-κB signaling pathway, a regulator of innate immunity (23). It has also been reported that molecular hydrogen treatment decreases fasting blood glucose levels, increases hepatic glycogen synthesis, and improves insulin sensitivity (23). Given these findings, we are interested in the effects of molecular hydrogen on TG and cholesterol regulation, which are implicated in the development of insulin resistance. Hydrogen-rich coral calcium (HRCC) capsules were certified by the Taiwan Food and Drug Administration (FDA) in 2020.^1^ We then investigated the safety and potential lipid-lowering effects of HRCC.

## 2. Materials and methods

### 2.1. Study design

The patients with metabolic syndrome were recruited from the outpatient department (OPD) in Min-Sheng General Hospital, Taiwan. Participants were screened to determine eligibility by doctors and underwent a series of tests involving questionnaires and examinations. In this study, the patients who met 3 or more of the following criteria were designated to have metabolic syndrome: (1) waist circumference: men ≥90 cm/women ≥80 cm, (2) systolic pressure ≥ 130 mmHg and/or diastolic pressure ≥ 85 mmHg or the use of antihypertensive drug, (3) fasting blood sugar level of 100 mg/dL or higher or use of antidiabetic medication, (4) fasting TG level ≥ 150 mg/dL or the use of lipid-lowering agent and (5) low HDL-C level: men <40 mg/dL/women <50 mg/dL (24). Consenting participants were allocated into 3 groups defined by their doses of HRCC capsules (low, *n* = 5; medium, *n* = 5; high, *n* = 6). The patients, who <18 years or unwilling to participate in this study were excluded. The patients received 1 (low), 3 (medium), or 6 (high) capsules of HRCC to take alongside conventional treatment daily for 4-week. The primary outcome was to measure the adverse effects/symptoms during this 4-week use of HRCC. The secondary outcome was to compare lipid profiles and other biomarkers changes including complete blood count (CBC), inflammatory biomarkers (erythrocyte sedimentation rate, ESR60’; high-sensitivity-CRP, hs-CRP), and haemoglobin A1c (HbA1C). The QOL was also checked before and after the use of HRCC. Patients were excluded from this study if serious adverse side effects/symptoms occurred. At the week 0, participants were instructed on the experimental procedure, and their consents were obtained. They received blood drawn and completed the pre-test including Brief Fatigue Inventory-Taiwan (BFI-T) and control status scale for diabetes (CSSD70) surveys. The participants also checked the changes of body weight during the 28 days. Finally, the participants had blood drawn and completed the post-test BFI-T and CSSD70 surveys. Research assistants provided the assistance and closely monitor the adverse effects/symptoms.

### 2.2. Materials

Hydrogen capsules (PURE HYDROGEN) were purchased from HoHo Biotech Co., Ltd. (Taipei, Taiwan). Each capsule contained 170 mg HRCC which contained 1.7*10^21 molecules of hydrogen (approximately 24 cups of 1,200 ppb/0.6 mM, 200 mL of hydrogen water). Therefore, the low dose contained 170 mg, the medium dose contained 510 mg, and the high dose contained 1,020 mg HRCC.

### 2.3. Measurements of adverse effects/symptoms

We measured any adverse effect/symptom up to 28 days, which were codified according to the Common Terminology Criteria for Adverse Events (NCI CTCAE v5.0). The measures included changes from baseline (at day 28) for the following: (1) routine blood parameters; (2) routine urine parameters; (3) responses captured by the BFI-T scale (6 questions), which is a 9-item, 11-point rating scale developed to assess subjective fatigue. The first three questions measure fatigue severity with 0 indicating “no fatigue” (total range: 0 to 100); and (4) responses captured by the CSSD70 questionnaire, in which the minimum score was 0 (most severe) and the maximum score was 2 (healthiest), total range: 0 to 22.

### 2.4. Statistical analysis

The patients with metabolic syndrome were enrolled in the study and provided data on body weight, TG, LDL-C, HDL-C, HbA1C, complete blood count, hs-CRP, ESR60’, BFI-T, and CSSD70. Data were expressed as mean and standard deviation (STD). Differences for which *p* < 0.05 were considered statistically significant. The analysis was conducted using paired-sample t tests and the statistical software Prism was used for statistical analyses.

## 3. Results

### 3.1. Demographics

A total of 16 patients who were confirmed to have metabolic syndrome completed this study (7 males, 9 females; mean age: 62 years; range: 32–80). The demographics were described in Table 1. None of the patients that were administered HRCC reported experiencing adverse effects or toxicity responses. A total 4 out of 16 patients reported improvements in sleep, and 4 out of 16 patients presented improvements in energy (Table 2).

**Table 1:** The demographic and baseline characteristics of the patients (*n* = 16).

	Male	Female	Total
Patients	7	9	16
Age, mean ± SD (years)	59.17	65.25	62.64
TG (mg/dL)	203	185.55	193.19
HDL-C (mg/dL)	42.86	49.55	46.63
LDL-C (mg/dL)	105.28	109.88	107.88
HbA1C (%)	7.26	6.64	6.91

**Table 2:** Qualitative responses reported by the patients (*n* = 16).

Number	Patient	Sex	Age	Dosage (capsules/day)	Qualitative response before treatment	Qualitative response after treatment	Weight change (kg)
1	HM012	F	32	6	Look to lose weight	Felt no specific changes	0
2	HM013	F	70	6	Have low platelets, high blood pressure, I cannot squat down. Knee joint surgery is needed, but due to low platelets, doctors do not recommend surgery.	Dizziness, nothing else	0
3	HM014	M	38	6	Diabetes, feet have very obvious pigmentation, the calf also throbbing. Taking medicine helps.	Felt no specific changes	−0.7
4	HM015	F	46	6	Breast cancer, low energy	Defecation is much improved and sleep is much better	0
5	HM017	F	71	6	Great improvement in mental and sleep quality	Sleep improved	0
6	HM018	M	58	6	Diabetes	My hands are still numb, and I do not feel anything else	0
7	HM006	M	71	3	Diabetes	Energy improved; wanted to continue treatment	−1
8	HM007	F	72	3	Eating will cause nausea. May need kidney dialysis, and have control over eating. Urine protein drugs. Tiredness every day.	Energy improved, and bowel movements were significantly smoother	−0.5
9	HM008	F	80	3	Often chest tightness, the heart is not comfortable	No show	0
10	HM009	M	N/A	3	Hard of hearing. The heart has 2–3 stents, calf pain occasionally.	Severe sleep disturbance and panic disorder. Taking capsules in the afternoon greatly improves sleep. The panic disorder no longer occurs, and the mood is much brighter.	0
11	HM010	M	53	3	It is not particularly uncomfortable, it is purely a matter of endocrine	No specific changes	2
12	HM001	F	75	1	Showed interest in HRCC	Felt but the condition was preexisted	1
13	HM003	F	80	1	Taking bone-guiding health products, diabetes medicine, very talkative, menopause	CSSD70 improved	0.8
14	HM004	M	63	1	A little hard to hear, hands are shaking	Energy improved in the first week after the treatment but did not feel specific changes after	0
15	HM005	M	68	1	Occasionally throbbing on the soles of the feet, tiredness, moods fluctuating	Energy improved; could not sleep well at night	0
16	HM016	F	68	1	Have diabetes, high blood pressure, cholesterol	Sleep is much better, can fall asleep deeply	0

### 3.2. Body weight

**Figure 1:**
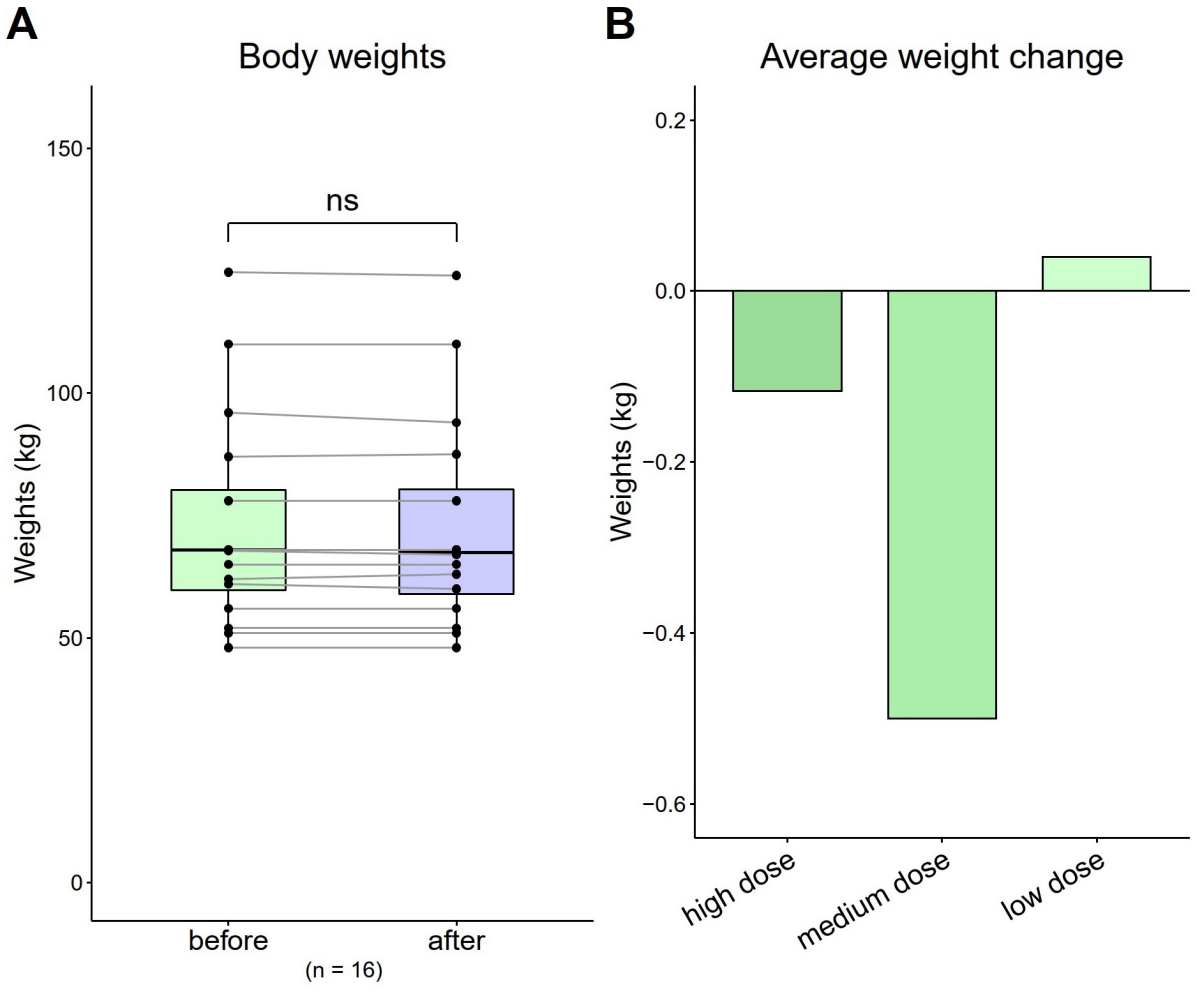
Total and average weight changes (*n* = 16). **(A)** Total body weight of all patients. **(B)** Average weight change by dose. Data are represented by a line plot and a bar graph (*p < 0.05; **p < 0.01; ***p < 0.001) and were analyzed using the paired *t* test.

The average weight of all patients before and after HRCC treatment was 73.91 kg (SD = 20.73) and 73.71 kg (SD = 20.53), respectively. No significant differences in body weight were observed [Figure 1A; *t*(16) = 0.57, *p* = 0.57]. The average weight of patients that received the high dose was 86.11 kg at baseline, which decreased to 86 kg after treatment (difference: −0.12 kg). The average weight of patients that received the medium dose was 69.4 kg at baseline, which decreased to 68.9 kg (difference: −0.5 kg). The average weight of patients that received the low dose was 63.76 kg at baseline, which decreased to 63.8 kg (difference: 0.04 kg) (Figure 1B).

### 3.3. Serum lipid profile (TG, HDL-C, LDL-C) and HbA1C

**Figure 2:**
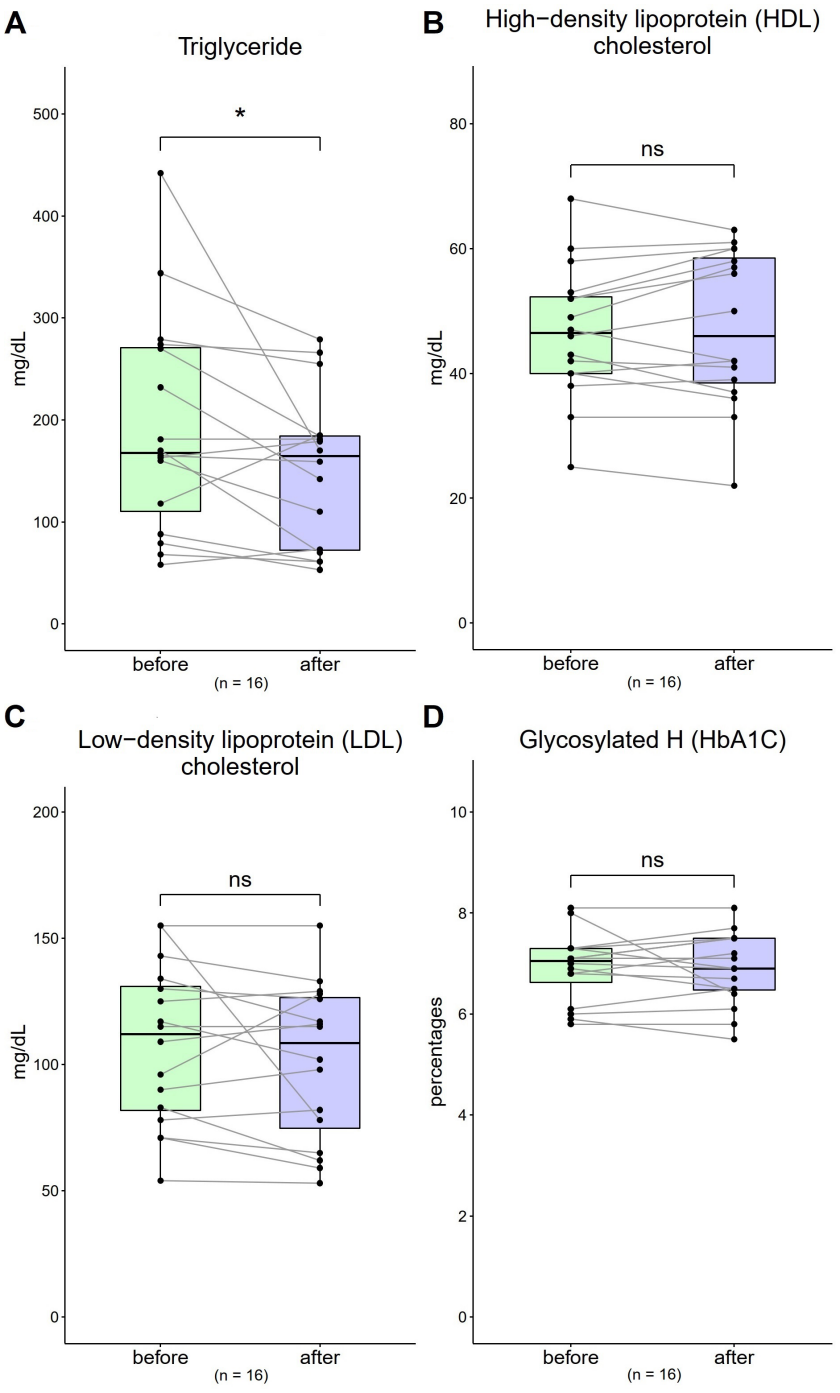
Changes in triglyceride (TG), high-density lipoprotein cholesterol (HDL-C), low-density lipoprotein cholesterol (LDL-C), and haemoglobin A1c (HbA1c) levels from 0 to 4 weeks (*n* = 16). **(A,C)** The normal ranges are <150 mg/dL and <130 mg/dL for TG and LDL-C, respectively. **(B)** The normal range for HDL-C is >40 mg/dL. **(D)** The normal range of HbA1c is 4.0–6.0%. Data are represented by line plots (*p < 0.05; **p < 0.01; ***p < 0.001) and were analyzed using the paired *t* test.

The TG value was 193.19 mg/dL (SD = 107.44) at baseline, which decreased to 151.75 mg/dL (SD = 45.27) after 4 weeks [Figure 2A; *t*(16) = 4.19, *p* = 0.04]. The HDL-C value was 46.63 mg/dL (SD = 10.71) at baseline, which increased to 47.31 mg/dL (SD = 12.36) after 4 weeks [Figure 2B; *t*(16) = 0.62, *p* = 0.54]. The LDL-C value was 107.88 mg/dL (SD = 31.49) at baseline, which decreased to 101.13 mg/dL (SD = 31.13) after 4 weeks [Figure 2C; *t*(16) = 1.20, *p* = 0.25]. The baseline HbA1c value was 6.91% (SD = 0.68) at baseline, which decreased to 6.88% (SD = 0.71) after 4 weeks [Figure 2D; *t*(16) = 0.34, *p* = 0.74].

### 3.4. Complete blood count (CBC)

**Figure 3:**
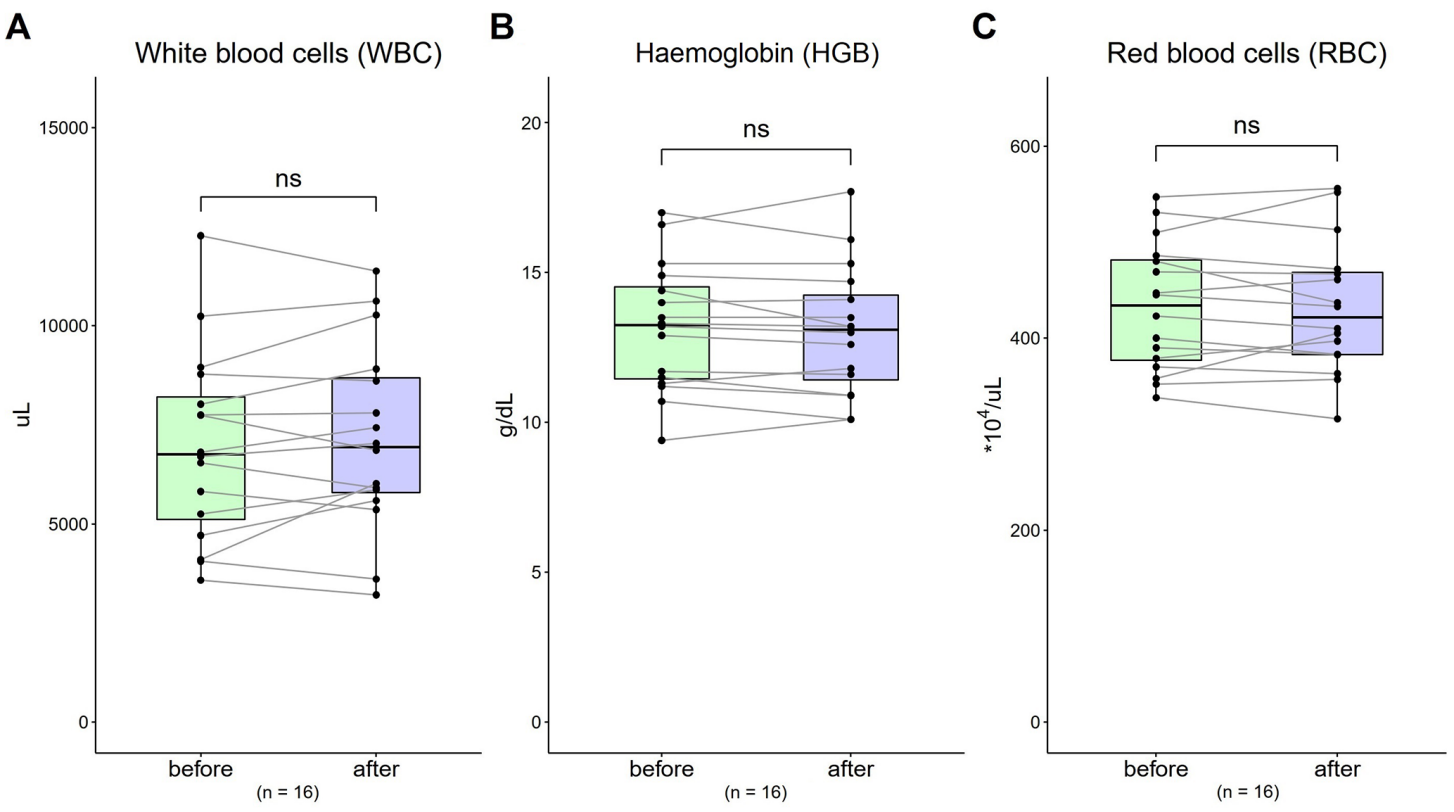
Changes in complete blood count (CBC) from 0 to 4 weeks (*n* = 16). **(A)** WBC = white blood cells (normal range: 3250 ~ 9,160/μL); **(B)** HGB = haemoglobin (normal range, males: 13.1 ~ 17.2; normal range, females: 11.0 ~ 15.2 g/dL); **(C)** RBC = red blood cells (normal range, males: 421 ~ 590; normal range, females: 378 ~ 525 *10^4/μL). Data are represented by line plots (*p < 0.05; **p < 0.01; ***p < 0.001) and were analyzed using the paired *t* test.

The WBC value was 6,957.5 μL (SD = 2,396.52) at baseline, and was 7,154.38 μL (SD = 2,361.75) after 4-week [Figure 3A; *t*(16) = 0.97, *p* = 0.35]. The HGB value at baseline was 13.18 g/dL (SD = 2.14), and was 13.05 g/dL (SD = 2.17) after 4-week [Figure 3B; *t*(16) = 0.92, *p* = 0.37]. The RBC value was 432.81*10^4 μL (SD = 66.49) at baseline, and was 431.56*10^4 μL (SD = 68.85) after 4-week [Figure 3C; *t*(16) = 0.21, *p* = 0.83].

### 3.5. Inflammatory biomarkers (ESR 60′ and hs-CRP)

**Figure 4:**
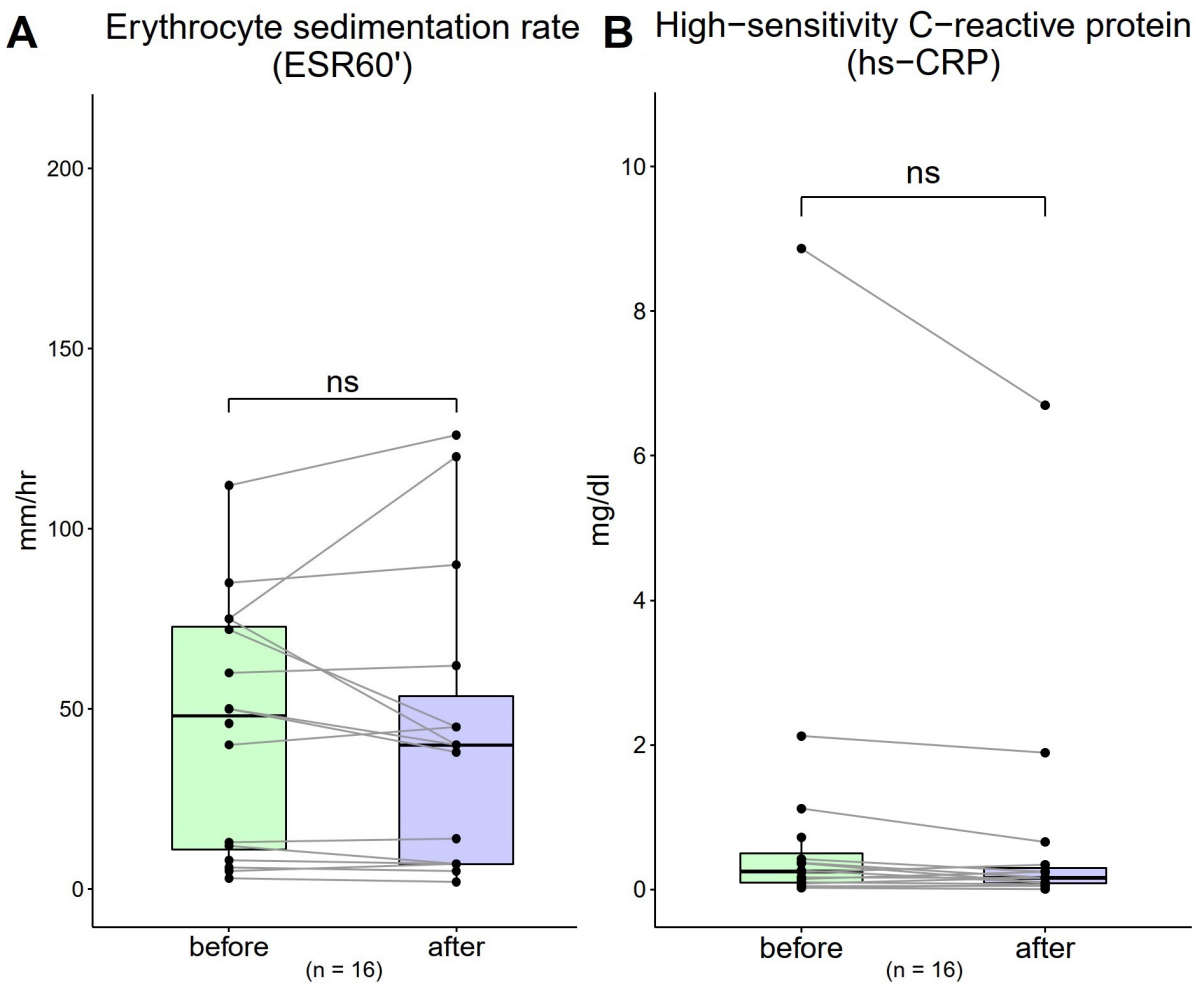
Changes in inflammatory biomarkers from 0 to 4 weeks (*n* = 16). **(A)** Erythrocyte sedimentation rate (ESR)-60 (normal range: males, <10; females, <15 mm/1 h) **(B)** high-sensitivity C-reactive protein (hs-CRP) (normal range: < 1 mg/dL). Data are represented as line plots (*p < 0.05; **p < 0.01; ***p < 0.001) and were analyzed using the paired *t* test.

The ESR 60′ value was 44.4 mm/dL (SD = 33.94) at baseline, which decreased to 43.2 mm/dL (SD = 40.79) after 4-week. A decreasing trend was observed; however, a one-sample t test showed no significant difference with treatment [Figure 4A; *t*(16) = 0.28, *p* = 0.79]. The hs-CRP value was 0.94 mg/dL (SD = 2.19) at baseline, which also decreased to 0.74 mg/dL (SD = 1.71) after 4-week. A decreasing trend was observed; however, a one-sample *t* test showed no significant difference with treatment [Figure 4B; *t*(16) = 1.45, *p* = 0.14].

### 3.6. Quality of life (QOL)

**Figure 5:**
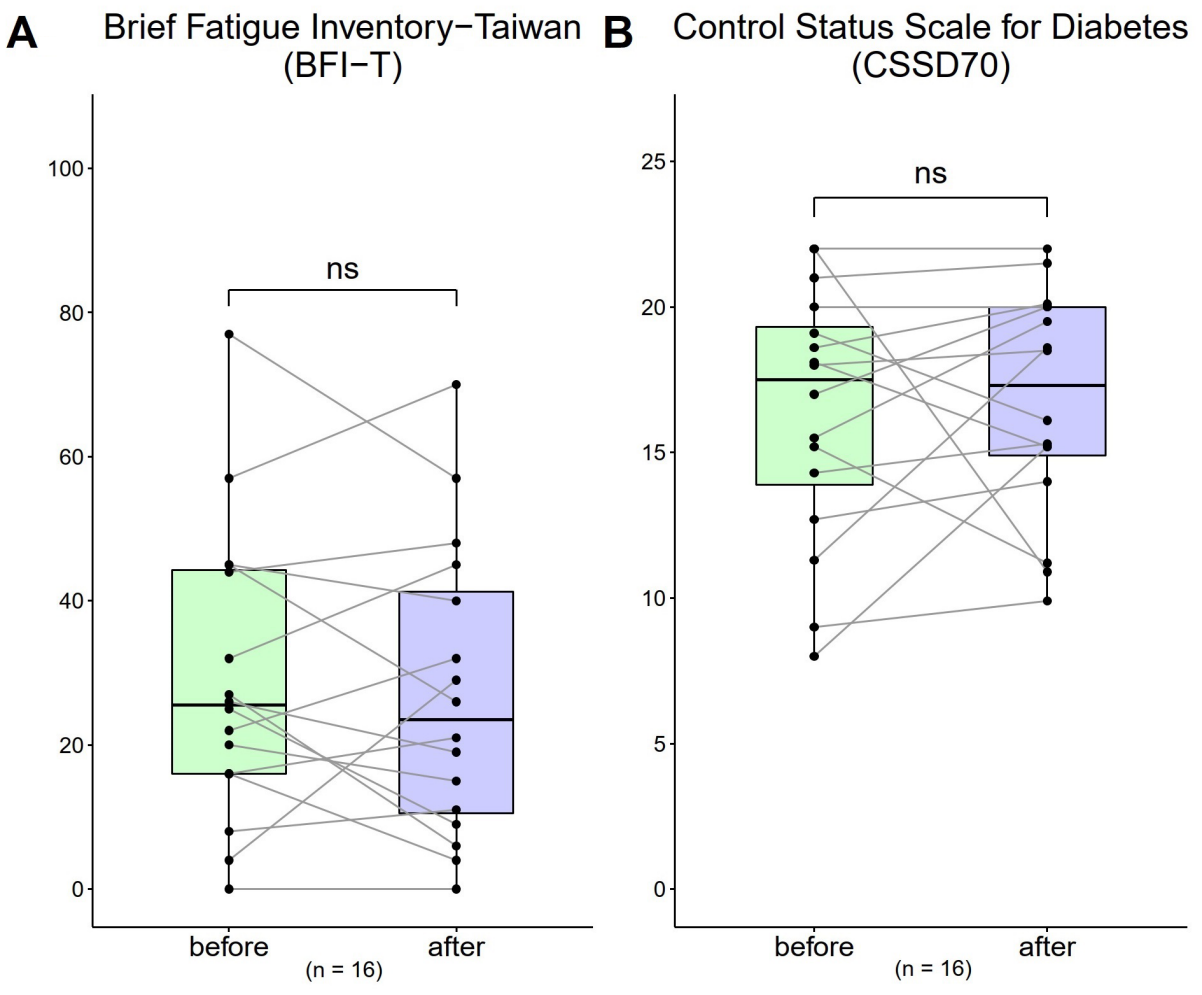
Changes in Brief Fatigue Inventory-Taiwan (BFI-T) and the control status scale for diabetes (CSSD70) scores from before to after treatment (*n* = 16). **(A)** The BFI-T consisted of 10 questions, each scored from 0 to 10, with 0 = most healthy and 10 = most severe (total range: 0 to 100); **(B)** The CSSD70 questionnaire consisted of 11 questions, each scored from 0 to 2 (total range: 0 to 22). Data are represented by line plots (*p < 0.05; **p < 0.01; ***p < 0.001) and were analyzed using the paired *t* test.

Both the results of BFI-T scale score and CSSD70 score showed no obvious changes before and after the 4-week use of HRCC (Figure 5).

## 4. Discussion

This study evaluated the potential TG-lowering effect of oral HRCC capsules in patients with metabolic syndrome. While the detailed mechanism is not well known, the significant decrease in TG could help explain the findings regarding the role of hydrogen in mediating TG by reducing excess oxidative stress. Future investigations should focus on the long-term efficacy of HRCC in patients with metabolic syndrome, especially the effects on HDL-C, LDL-C, and HbA1C. Larger sample sizes and longer experimental periods are needed to establish clinical efficacy and to determine the optimal dose.

The short-term safety endpoint of this study, which was used to test the safety of oral HRCC capsules at different doses, was met. After 1 month of treatment, HRCC was not associated with any patient-reported acute adverse effects. Most of the biomarkers measured in this study, including plasma or urinary biomarkers, WBC, HGB, RBC, HDL-C, LDL-C, HbA1C, inflammatory indicators such as CRP, eosinophils, and ESR, and body weight did not change significantly after treatment, except for TG, which decrease significantly after treatment. In this study, BFI-T and health in type 2 diabetic patients (CSSD70) did not significantly change after the use of HRCC. Notably, the baseline CSSD70 score was 16.36 (out of the maximum of 22), which is considered high and an indication of positive health status in diabetic patients. The baseline BFI-T was 29 (out of the maximum of 100), which is considered mild. Diabetes is often associated with fatigue and dizziness (25). Signs such as increased thirst, frequent urination, urinary incontinence, numbness of the hands and feet, calf and foot pain are commonly associated with diabetes (26). Future research should be done in a larger sample over a longer time to understand the effects of HRCC capsules on the BFI-T and CSSD70. Qualitative indicated that 4 out of 16 patients experienced improvements in sleep and 4 out of 16 patients experienced improved energy, findings that require further investigation. H_2_ gas has been shown to have the potential to regulate mitochondrial energy metabolism (27). Kamimura et al. (15) reported that prolonged consumption of HRW significantly altered fat and body weight in db/db obese mice by stimulating energy metabolism. Hydrogen treatment has also been documented to have anti-fatigue effects, and boost running performance and torso strength in healthy adults given hydrogen inhalation therapy (28).

Inhalation of hydrogen gas is the most straightforward therapeutic method and has been used worldwide. Inhalation of H_2_ ensures a long retention time and high dose in the body. Inhaled hydrogen diffuses through the alveoli into the plasma and is transported throughout the body through the blood. Nevertheless, the different administration methods of hydrogen may have different effects (29). A pharmacokinetic study of a single inhalation of hydrogen gas in pigs showed that the hydrogen level in the carotid artery peaked immediately after breath holding, and it dropped to 1/40 of the peak value 3 min later. The peak hydrogen concentration in venous blood was much lower than that in arterial blood, which indicated that hydrogen is not simply diffused but diffuses while being carried through the blood stream (29). Molecular hydrogen/saline injection is a method that can rapidly supply a large amount of hydrogen to the body and allows direct application of hydrogen to the affected area. However, this method is invasive and difficult for patients to accept, and it has the potential risk of cross-infection. In addition, it could be dangerous if hydrogen is injected directly into the skin or vein. Thus, the different administration routes of hydrogen need to be considered according to the needs of the patient to guarantee the most benefit. A previous study found that preinhalation of hydrogen gas could protect against caerulein-induced acute pancreatitis in mice by inhibiting inflammation and oxidative stress in the early stage (30). Some clinical trials have also demonstrated that hydrogen gas inhalation treatment is safe and effective in patients with chronic obstructive pulmonary disease (COPD) and asthma (31, 32). Therapeutic effects in patients with oxidative stress owing to cardiac arrest and inflammation have also been shown (33, 34). Recently, to confront the coronavirus disease-2019 (COVID-19) pandemic caused by severe acute respiratory syndrome coronavirus 2 (SARS-CoV-2), starting with the Chinese Clinical Guidance (7th edition) for COVID-19 Pneumonia Diagnosis and Treatment issued by the China National Health Commission, the inhalation of oxygen mixed with hydrogen gas (66.6% hydrogen gas and 33.3% oxygen) has been recommended given the significant role of hydrogen in ameliorating lung function decline, emphysema, and inflammation among patients with pulmonary diseases (35). In summary, the therapeutic effects of hydrogen have been highlighted in various human diseases or functional disorders. Future trials should assess self-reported lifestyle changes, provide a historical control, and compare a drug treatment group with a control group to clarify the effects of oral HRCC capsules.

To the best of our knowledge, this is the first human study investigating the safety and therapeutic efficacy of oral molecular hydrogen capsules in patients with metabolic syndrome. One strength of this study is the comprehensive evaluation of serum biomarkers in response to different doses. Unfortunately, the sample size, sample size per dose group, and short-term use of the current cohort were limiting factors. In addition, there was a large age difference between the groups. It will be important to compare the results between the different sexes. The long-term therapeutic effects of HRCC capsules and the influence of the dose in large populations should be assessed in future studies.

## 5. Conclusion

This first human clinical trial evaluating HRCC capsules in patients with metabolic syndrome demonstrated their safety and a potential therapeutic effect from lowering TG. No adverse effects were observed. Findings regarding CBC, HbA1C, hs-CRP and ESR-60, a fatigue indicator (BFI-T), and the CSSD70 questionnaire all indicate that HRCC did not cause obvious adverse effects. Future studies with a larger sample size are required to perform a long-term safety evaluation and assess HRCC efficacy in metabolic syndrome, such as high blood pressure, elevated fasting glucose levels, and low HDL-C. After further study, the therapeutic effects of HRCC capsules in combination with medications such as statins and niacin could help to prevent and treat metabolic syndrome or other metabolic diseases.

## Data availability statement

The original contributions presented in the study are included in the article/Supplementary material, further inquiries can be directed to the corresponding author.

## Ethics statement

The studies involving human participants were reviewed and approved by the research protocol was approved by the Institution Review Board (IRB) of Min-Sheng General Hospital (MSIRB). The approval number is 2021007-A. The patients/participants provided their written informed consent to participate in this study.

## Author contributions

K-YW, H-YC, J-RC, T-YW, P-JH, and W-WC performed the material preparation, data collection, and analysis. S-HC, J-RC, P-JH, W-CH, K-YW, C-FC, and M-CS wrote the first draft of the manuscript. F-CL, P-JH, and FD supervised the manuscript. All authors contributed to the study conception and design, commented on all versions of the manuscript, and read and approved the final manuscript.

## Funding

This article was supported by the HoHo Biotech (ho202102040).

## Conflict of interest

FD, J-RC, K-YW, C-FC, H-YC, and W-CH were employed by the HoHo Biotech Co., Ltd. T-YW was employed by HoGo Force Co., Ltd.

The authors declare that this study received funding from HoHo Biotech. The funder had the following involvement in the study: experiment design, data collection, and the decision to publish.

## Publisher’s note

All claims expressed in this article are solely those of the authors and do not necessarily represent those of their affiliated organizations, or those of the publisher, the editors and the reviewers. Any product that may be evaluated in this article, or claim that may be made by its manufacturer, is not guaranteed or endorsed by the publisher.

## Figures and Tables

**Table:** 

HRCC	Hydrogen-rich coral calcium
TG	Triglyceride. A TG test is a blood test that measures the amount of fat in the blood called TG. High TG may increase the risk of a heart attack or stroke
T2DM	Type 2 diabetes
Cardiovascular diseases (CVD)	Cardiovascular diseases
QOL	Quality of life
HDL-C	High-density lipoprotein cholesterol, often referred to as “good” cholesterol
LDL-C	Low-density lipoprotein cholesterol, often called the “bad” cholesterol because it adheres to the walls of blood vessels, increasing the probability of developing health problems such as heart attack or stroke
TNFα	Tumor necrosis factor α (TNFα). An inflammatory cytokine that is produced by macrophages/monocytes during acute inflammation and is responsible for a diverse range of signaling events within cells, leading to necrosis or apoptosis
IL-6	Interleukin-6, a cytokine that plays a central role in host defence due to its wide range of immune and haematopoietic activities
ESR	Erythrocyte sedimentation rate is the rate at which red blood cells in anticoagulated whole blood descend in a standardized tube over a period of 1 h
CRP	C-reactive protein. A wide variety of inflammatory conditions can cause elevated CRP levels
WBC	White blood cells
BFI-T	Brief Fatigue Inventory-Taiwan (BFI-T)
CSSD70	Control status scale for diabetes questionnaire. Each question is scored with a minimum value of 0 (the most severe), and a maximum value of 2 (the most healthy). Total range from 0 to 22
RBC	Red blood cells
MCH	Mean corpuscular haemoglobin
MCV	Mean corpuscular volume
MCHC	Mean corpuscular haemoglobin concentration
HbA1C	The haemoglobin A1c test measures the amount of blood sugar (glucose) attached to haemoglobin
CHOL	A blood test that can measure the amount of cholesterol and triglycerides in the blood
hs-CRP	High-sensitivity CRP
HGB	The haemoglobin test measures the quantity of haemoglobin contained in the red blood cells

## Footnotes

^1^ https://consumer.fda.gov.tw/Food/CapsuleAuditQueryDetail.aspx?nodeID=165&id=1096000144

## References

[1] Grundy SM. Advancing drug therapy of the metabolic syndrome. Nat Rev Drug Discov. (2009) 8:341. doi: 10.1038/nrd2894 16582875

[2] Falkner B Cossrow ND. Prevalence of metabolic syndrome and obesity-associated hypertension in the racial ethnic minorities of the United States. Curr Hypertens Rep. (2014) 16:449. doi: 10.1007/s11906-014-0449-5, PMID: 24819559 PMC4083846

[3] Iyen B Akyea RK Weng S Kai J Qureshi N. Statin treatment and LDL-cholesterol treatment goal attainment among individuals with familial hypercholesterolaemia in primary care. Open Heart. (2021) 8:e001817. doi: 10.1136/openhrt-2021-001817, PMID: 34702779 PMC8549660

[4] Wagh A Stone NJ. Treatment of metabolic syndrome. Expert Rev Cardiovasc Ther. (2004) 2:213–28. doi: 10.1586/14779072.2.2.213 15151470

[5] Wierzbicki AS Viljoen A. Fibrates and niacin: is there a place for them in clinical practice? Expert Opin Pharmacother. (2014) 15:2673–80. doi: 10.1517/14656566.2014.972365, PMID: 25318657

[6] Wilcox G. Insulin and insulin resistance. Clin Biochem Rev. (2005) 26:19–39. PMID: 16278749 PMC1204764

[7] Suiter C Singha SK Khalili R Shariat-Madar Z. Free fatty acids: circulating contributors of metabolic syndrome. Cardiovasc Haematol Agents Med Chem. (2018) 16:20–34. doi: 10.2174/1871525716666180528100002, PMID: 29804539

[8] Alberti KG Zimmet P Shaw J. Metabolic syndrome--a new worldwide definition. A consensus statement from the international diabetes federation. Diabet Med. (2006) 23:469–80. doi: 10.1111/j.1464-5491.2006.01858.x 16681555

[9] Lau DC Yan H Dhillon B. Metabolic syndrome: a marker of patients at high cardiovascular risk. Can J Cardiol. (2006) 22:85B–90B. doi: 10.1016/s0828-282x(06)70992-8, PMID: 16498518 PMC2780835

[10] Ridker PM Buring JE Cook NR Rifai N. C-reactive protein, the metabolic syndrome, and risk of incident cardiovascular events: an 8-year follow-up of 14 719 initially healthy American women. Circulation. (2003) 107:391–7. doi: 10.1161/01.cir.0000055014.62083.05, PMID: 12551861

[11] Alende-Castro V Alonso-Sampedro M Fernández-Merino C Sánchez-Castro J Sopeña B Gude F. C-reactive protein versus erythrocyte sedimentation rate: implications among patients with no known inflammatory conditions. J Am Board Fam Med. (2021) 34:974–83. doi: 10.3122/jabfm.2021.05.210072, PMID: 34535522

[12] Amini M Bashirova D Prins BP Corpeleijn E Bruinenberg M Franke L. Eosinophil count is a common factor for complex metabolic and pulmonary traits and diseases: the lifelines cohort study. PLoS One. (2016) 11:e0168480. doi: 10.1371/journal.pone.0168480, PMID: 27978545 PMC5158313

[13] Zhang Y Tan S Xu J Wang T. Hydrogen therapy in cardiovascular and metabolic diseases: from bench to bedside. Cell Physiol Biochem. (2018) 47:1–10. doi: 10.1159/000489737, PMID: 29763888

[14] Masuch A Budde K Kastenmüller G Artati A Adamski J Völzke H. Metabolic signature associated with parameters of the complete blood count in apparently healthy individuals. J Cell Mol Med. (2019) 23:5144–53. doi: 10.1111/jcmm.14383, PMID: 31215770 PMC6652895

[15] Kamimura N Nishimaki K Ohsawa I Ohta S. Molecular hydrogen improves obesity and diabetes by inducing hepatic FGF21 and stimulating energy metabolism in db/db mice. Obesity. (2011) 19:1396–403. doi: 10.1038/oby.2011.6, PMID: 21293445

[16] Kajiyama S Hasegawa G Asano M Hosoda H Fukui M Nakamura N. Supplementation of hydrogen-rich water improves lipid and glucose metabolism in patients with type 2 diabetes or impaired glucose tolerance. Nutr Res. (2008) 28:137–43. doi: 10.1016/j.nutres.2008.01.008 19083400

[17] Song G Li M Sang H Zhang L Li X Yao S. Hydrogen-rich water decreases serum LDL-cholesterol levels and improves HDL function in patients with potential metabolic syndrome. J Lipid Res. (2013) 54:1884–93. doi: 10.1194/jlr.M036640, PMID: 23610159 PMC3679390

[18] LeBaron TW Singh RB Fatima G Kartikey K Sharma JP Ostojic SM. The effects of 24-week, high-concentration hydrogen-rich water on body composition, blood lipid profiles and inflammation biomarkers in men and women with metabolic syndrome: a randomized controlled trial. Diabetes Metab Syndr Obes. (2020) 13:889–96. doi: 10.2147/dmso.s240122, PMID: 32273740 PMC7102907

[19] Adzavon YM Xie F Yi Y Jiang X Zhang X He J. Long-term and daily use of molecular hydrogen induces reprogramming of liver metabolism in rats by modulating NADP/NADPH redox pathways. Sci Rep. (2022) 12:3904. doi: 10.1038/s41598-022-07710-6, PMID: 35273249 PMC8913832

[20] Sugai K Tamura T Sano M Uemura S Fujisawa M Katsumata Y. Daily inhalation of hydrogen gas has a blood pressure-lowering effect in a rat model of hypertension. Sci Rep. (2020) 10:20173. doi: 10.1038/s41598-020-77349-8, PMID: 33244027 PMC7692487

[21] Kishimoto Y Kato T Ito M Azuma Y Fukasawa Y Ohno K. Hydrogen ameliorates pulmonary hypertension in rats by anti-inflammatory and antioxidant effects. J Thorac Cardiovasc Surg. (2015) 150:645–54.e3. doi: 10.1016/j.jtcvs.2015.05.052, PMID: 26095621

[22] Dixon BJ Tang J Zhang JH. The evolution of molecular hydrogen: a noteworthy potential therapy with clinical significance. Med Gas Res. (2013) 3:10. doi: 10.1186/2045-9912-3-10, PMID: 23680032 PMC3660246

[23] Ming Y Ma QH Han XL Li HY. Molecular hydrogen improves type 2 diabetes through inhibiting oxidative stress. Exp Ther Med. (2020) 20:359–66. doi: 10.3892/etm.2020.8708, PMID: 32537002 PMC7291681

[24] Huang PH Lu YW Tsai YL Wu YW Li HY Chang HY. Taiwan lipid guidelines for primary prevention. J Formos Med Assoc. (2022) 121:2393–407. doi: 10.1016/j.jfma.2022.05.010, PMID: 35715290

[25] Kalra S Sahay R. Diabetes fatigue syndrome. Diabetes Ther. (2018) 9:1421–9. doi: 10.1007/s13300-018-0453-x, PMID: 29869049 PMC6064586

[26] Golbidi S Laher I. Bladder dysfunction in diabetes mellitus. Front Pharmacol. (2010) 1:136. doi: 10.3389/fphar.2010.00136, PMID: 21833175 PMC3153010

[27] Tian Y Zhang Y Wang Y Chen Y Fan W Zhou J. Hydrogen, a novel therapeutic molecule, regulates oxidative stress, inflammation, and apoptosis. Front Physiol. (2021) 12:789507. doi: 10.3389/fphys.2021.789507, PMID: 34987419 PMC8721893

[28] Javorac D Stajer V Ratgeber L Betlehem J Ostojic S. Short-term H2 inhalation improves running performance and torso strength in healthy adults. Biol Sport. (2019) 36:333–9. doi: 10.5114/biolsport.2019.88756, PMID: 31938004 PMC6945053

[29] Cole AR Sperotto F DiNardo JA Carlisle S Rivkin MJ Sleeper LA. Safety of prolonged inhalation of hydrogen gas in air in healthy adults. Crit Care Explor. (2021) 3:e543. doi: 10.1097/CCE.0000000000000543, PMID: 34651133 PMC8505337

[30] Li K Yin H Duan Y Lai P Cai Y Wei Y. Preinhalation of hydrogen-rich gases protect against caerulein-induced mouse acute pancreatitis while enhance the pancreatic Hsp60 protein expression. BMC Gastroenterol. (2021) 21:178. doi: 10.1186/s12876-021-01640-9, PMID: 33874887 PMC8056676

[31] Wang ST Bao C He Y Tian X Yang Y Zhang T. Hydrogen gas (XEN) inhalation ameliorates airway inflammation in asthma and COPD patients. QJM. (2020) 113:870–5. doi: 10.1093/qjmed/hcaa164, PMID: 32407476 PMC7785302

[32] Zheng ZG Sun WZ Hu JY Jie ZJ Xu JF Cao J. Hydrogen/oxygen therapy for the treatment of an acute exacerbation of chronic obstructive pulmonary disease: results of a multicentre, randomized, double-blind, parallel-group controlled trial. Respir Res. (2021) 22:149. doi: 10.1186/s12931-021-01740-w, PMID: 33985501 PMC8120708

[33] Ostojic SM Vukomanovic B Calleja-Gonzalez J Hoffman JR. Effectiveness of oral and topical hydrogen for sports-related soft tissue injuries. Postgrad Med. (2014) 126:187–95. doi: 10.3810/pgm.2014.09.2813, PMID: 25295663

[34] Tamura T Suzuki M Hayashida K Kobayashi Y Yoshizawa J Shibusawa T. Hydrogen gas inhalation alleviates oxidative stress in patients with postcardiac arrest syndrome. J Clin Biochem Nutr. (2020) 67:214–21. doi: 10.3164/jcbn.19-101 33041520 PMC7533855

[35] Guan WJ Chen RC Zhong NS. Strategies for the prevention and management of coronavirus disease 2019. Eur Respir J. (2020) 55:2000597. doi: 10.1183/13993003.00597-2020 32217658 PMC7098484

